# Gaze Cuing Effects in Peripheral Vision

**DOI:** 10.3389/fpsyg.2019.00708

**Published:** 2019-04-04

**Authors:** Takemasa Yokoyama, Yuji Takeda

**Affiliations:** ^1^ Automotive Human Factors Research Center, National Institute of Advanced Industrial Science and Technology, Tsukuba, Japan; ^2^ Faculty of Human Science, University of Tsukuba, Tsukuba, Japan

**Keywords:** gaze cuing effect, peripheral vision, attention, gaze perception, visual angle

## Abstract

When we see another person’s gaze, spatial attention shifts toward the gaze direction. Thus, a gaze perceiver can more quickly respond to a forthcoming target when it appears in a direction of a gaze giver than when it does not. This phenomenon is termed the *gaze cuing effect*. Previous studies have investigated the gaze cuing effect only in foveal vision; hence, it remains unclear whether the gaze cuing effect is induced when a face is presented in peripheral vision. This is an important issue because in our daily lives we communicate not only with people in front of us but also with those in our periphery. To tackle this question, we manipulated vertically aligned locations of a facial stimulus (i.e., a face stimulus appeared above or below the center fixation) and tested the extent to which a gaze cuing effect, conveyed by gaze shifts of another, is observed in the periphery. The facial stimulus was located 0, ±2.5, ±5.0, and ±7.5° of the visual angle from the center of the display, and a target was presented 5.6° to the left or right of the center of the display. In Experiment 1, when participants responded to the location of an abrupt onset of a target (i.e., localization task), we observed significant gaze cuing effects when a facial stimulus was located 0, ±2.5, and ±5.0°, but not ±7.5°. In Experiment 2, we replicated the findings in Experiment 1 if participants pressed a key only when a target appeared (i.e., detection task). In Experiment 3, we used adjusted sizes of facial images based on the cortical representations and manipulated eye directions of the facial images oriented toward the possible target locations; it resulted in enlarged effective field of view for gaze cuing effects. The study reveals that gaze cuing effects can appear even in peripheral vision and within a vertical distance of 5.0° of the visual angles, but the effective field of view is expanded when the facial image is adjusted based on the cortical representations, and eye gaze directly looks at the possible target locations.

## Introduction

People receive a wealth of social information from the gaze of others, information that we use to facilitate social interactions. Furthermore, people can infer what others favor and where their interest lies. Therefore, people habitually pay attention to another’s gaze in order to anticipate the mental state of a gaze givers, i.e., his/her thoughts and desires (Baron-Cohen, 1995). Because the information from the gaze of another person allows us to determine appropriate behavior in relation to the gaze giver, such social abilities facilitate social interactions. Thus, gaze perception plays a critical role in social situations.

Gaze perception facilitates cognitive processes, such as attention as well as social interactions. For example, if the gaze direction of another indicates a rightward direction, then the spatial attention of the gaze perceiver typically shifts to align with the gaze direction of the gaze giver (Friesen and Kingstone, 1998; Driver et al., 1999; Langton et al., 2000). This phenomenon is termed a gaze cuing effect (Frischen et al., 2007; Birmingham and Kingstone, 2009). To examine this phenomenon, a modified Posner paradigm (i.e., gaze cuing paradigm) is usually enlisted. Thus, if a target is presented in the direction indicated by gaze, participants can make a response to the target more quickly. The gaze cuing effect is induced even when a gaze direction does not accurately predict a target location (Friesen and Kingstone, 1998). In addition, this automatic attentional orienting is intact even when other functions, such as working memory, also consume resources for spatial representations (Yokoyama et al., 2019). Because the gaze of another individual modulates automatic attentional orienting despite adverse situations, the gaze cue is a powerful visual stimulus to modulate spatial attention.

Although there are numerous studies of gaze perception involving foveal vision, very little is known about gaze perception involving peripheral vision. In a gaze discrimination task, Loomis et al. (2008) manipulated locations of a facial stimulus and found that participants could discriminate gaze directions of the facial stimulus to the extent of 4° visual angles. Also, studies of Palanica and Itier (2014, 2017) revealed that accurate discrimination of gaze directions (e.g., left, right, up, down) covers up to 6° of horizontal and vertical eccentricities. In general, peripheral vision decreases spatial resolution of a visual stimulus (Chung et al., 1998; Nasanen and O’Leary, 1998), but Florey et al. (2015) found that poor gaze discrimination in peripheral vision is not the result of decreased spatial resolution, but rather it is due to other factors, such as crowding and prior information for gaze or head directions. In our previous study, participants could discriminate between direct and averted gaze, but not between leftward and rightward gaze, when their attention was allocated to the central letter discrimination task (Yokoyama et al., 2014). Although some studies have addressed gaze discrimination in peripheral vision, it remains unclear whether a gaze cuing effect occurs beyond foveal vision. When humans interact within a group, they must rely on peripheral vision for perceiving gaze of others, i.e., all group members are not always directly in front of a viewer. Hence, it is conceivable that the human visual system enables accurate gaze discrimination in peripheral vision. Similar to gaze discrimination, gaze cuing effects are also important in social interactions in which people provide information on future desires and interests through their eye movements.

The aim of this study was to ascertain whether gaze cuing effects would occur beyond foveal vision. To tackle this issue, we quantitatively manipulated location eccentricities of a facial image to measure the gaze cuing effect. Because a target appears at the side of a facial stimulus in a gaze cuing task (Friesen and Kingstone, 1998; Driver et al., 1999; Yokoyama et al., 2012), we manipulated vertical eccentricities. We prepared ±7.5, ±5.0, ±2.5°, or 0° (the center) from the center of the display (+ means upward; − means downward), and the facial stimulus was located at one of the seven possible locations. In Experiment 1, we conducted a localization task; participants pressed one of two keys corresponding to the location of an abrupt onset of a target. In Experiment 2, we conducted a detection task; in this case, participants pressed a key only when a target appeared. The latter aimed to replicate the Experiment 1 findings without contamination from the stimulus-response compatibility effect. In Experiment 3, we examined two additional factors: the effective field of view regarding the gaze cuing effect and the effect of accurate gaze directions to a target. To this end, the facial stimuli were adjusted based on the cortical representations, and eye gaze of the face stimuli directly looked at the possible target location.

## Experiment 1

### Method

#### Participants

Twenty-three paid volunteers (12 female, age range: 18–32) participated in Experiment 1. They provided written informed consent, as approved by the institutional review board of the National Institute of Advanced Industrial Science and Technology. All participants had normal or corrected-to-normal acuity of vision, and all were naïve to the purposes of this experiment.

### Apparatus

Visual stimuli were displayed on the ASUS ROG SWIFT PG258Q LCD display of 1,920 × 1,080 pixels (refresh rate was 120 Hz). Visual display and data collection were controlled using the Psychophysics Toolbox of MATLAB (Brainard, 1997; Pelli, 1997) on Microsoft Windows 7. Participants were tested individually in a darkened room, and the viewing distance was approximately 57 cm.

### Visual Stimuli and Procedure

**Figure 1:**
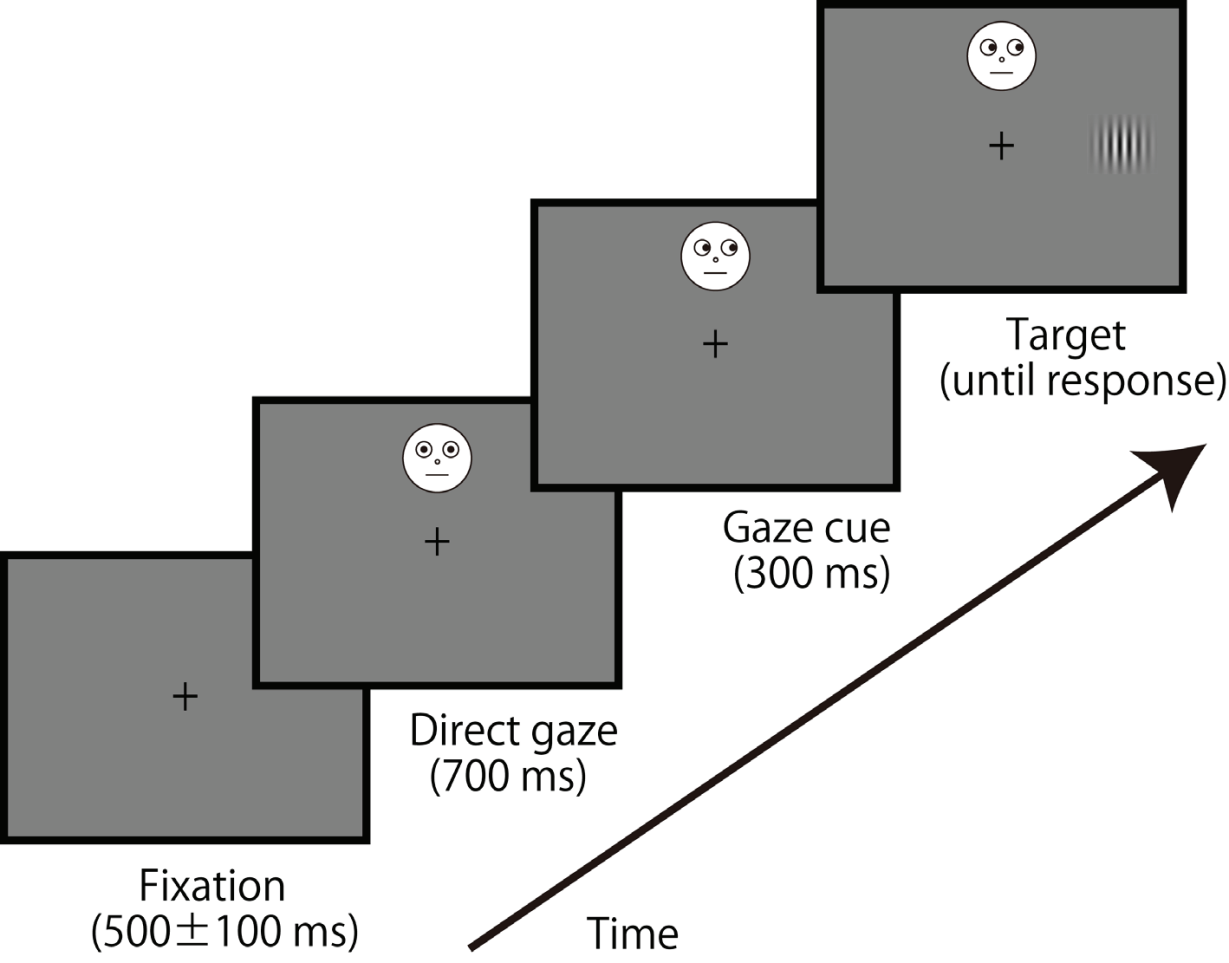
An example of the sequence of events in a typical trial. The face stimulus was presented up to ±7.5° of the visual angle (above and below) of vertical eccentricities, but the target was presented horizontally centered in the display.

We used a schematic face (2.3° × 2.3°); an example of the stimulus is shown in Figure 1. The central fixation was presented in 500 ± 100 ms, followed by a schematic face with direct gaze lasting 700 ms. Positions of the facial stimulus were located at ±7.5, ±5.0, ±2.5°, or 0° from the center of the display (0° was display center, and plus refers a locus above center with a minus signal representing below this center). Because eyes were positioned at a top of a facial image, those positions (±7.5, ±5.0, ±2.5, and 0°) matched the eye position. After eye gaze of the facial stimulus indicated a left or right for 300 ms (i.e., gaze cue), a target Gabor patch (size: 1.4° × 1.4°, spatial frequency: 3.2 c/d, Michelson contrast: 0.625, orientation: vertical) was presented 5.6° to the left or right of the center of the display until a response was made. Gaze direction of the face did not predict the target location, and participants were instructed about the manipulation before starting the experiment. Participants pressed the 1 (left) or 2 (right) key with their right hand as quickly and accurately as possible to judge the target location (i.e., a localization task) while they tried to ignore the gaze direction of the facial stimulus. Congruent (gaze direction and target location were identical) and incongruent (gaze direction and target location differed) conditions were randomly intermixed in a block. Also, the seven locations of the facial stimulus were randomly intermixed in a block. Twelve blocks of 84 trials were employed [total 1,008 trials: 72 trials × congruency (2) × location (7)], preceded by 14 practice trials.

### Results

**Figure 2:**
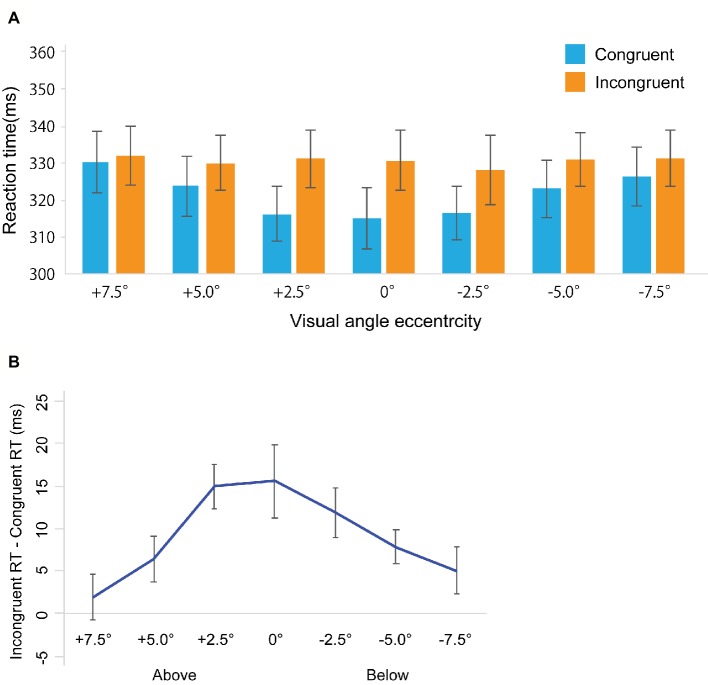
Results in Experiment 1. **(A)** Mean of (individual) median reaction time (ms). **(B)** The results of the magnitude of the gaze cuing effect (incongruent RT-congruent RT). The error bars represent standard error of mean.

Error trials were removed from further analysis (Total: 1.56%, congruent trials: 1.17%, incongruent trials: 1.95%). We used the median of collected reaction time data to examine gaze cuing effects as computed for every participant in each condition from the remaining data (Driver et al., 1999; Ristic et al., 2002; Burton et al., 2009; Ivanoff and Saoud, 2009; Marotta et al., 2013; Bobak and Langton, 2015) and computed for every participant in each condition from the remaining data. Figure 2A shows RTs in the gaze cuing task, and Figure 2B shows gaze cuing effects (RTs in the incongruent-congruent conditions) on RTs. We performed 2 *Congruency* (congruent, incongruent) × 7 *Location* (+7.5, +5.0, 2.5, 0, −2.5, −5.0, −7.5°) repeated measures analysis of variance (ANOVA). We found the main effect of *Congruency* [*F*_(1, 22)_ = 30.971, *p* < 0.001, $\eta_{p}^{2}$ = 0.421] and *Location* [*F*_(1, 22)_ = 5.076, *p* < 0.001, $\eta_{p}^{2}$ = 0.203]. There was also a significant interaction between *Congruency* and *Location* [*F*_(1, 22)_ = 5.037, *p* < 0.001, $\eta_{p}^{2}$ = 0.213]. To further assess the interaction between *Congruency* and *Location*, a simple main effect analysis was performed with a Bonferroni correction. This yielded significant differences between congruent and incongruent conditions in +5.0° (*t*_22_ = 2.272, *p* < 0.05, *d* = 0.16), +2.5° (*t*_22_ = 5.579, *p* < 0.001, *d* = 0.40), 0° (*t*_22_ = 3.36, *p* < 0.005, *d* = 0.36), −2.5° (*t*_22_ = 4.011, *p* < 0.001, *d* = 0.34), and −5° conditions (*t*_22_ = 3.721, *p* < 0.005, *d* = 0.21). In addition, we found a simple main effect of *Location* in the congruent condition [*F*_(1, 6)_ = 11.667, *p* < 0.001, $\eta_{p}^{2}$ = 0.347] but not in the incongruent conditions [*F*_(1, 6)_ = 0.458, *p* = 0.839, $\eta_{p}^{2}$ = 0.020].

### Discussion

We found that the congruent RTs were significantly faster than the incongruent RTs (i.e., consistent with the gaze cuing effect) beyond foveal vision. In addition, the gaze cuing effect persisted when a face was located a ±5.0° visual angle. Furthermore, we found face location effects only in the congruent condition.

In Experiment 1, we conducted the localization task in which participants were required to press one of two keys corresponding to the target location. Because cue directions and responses were consistent in the congruent condition, the stimulus-response compatibility might have been confounded in the results in Experiment 1. To rule out the possibility, in Experiment 2, we used a detection task in which participants were required to press a key only when a target appeared irrespective of the target location.

## Experiment 2

### Method

The method in Experiment 2 was identical to Experiment 1, with the exception of the following details.

### Participants and Procedure

Twenty-one paid volunteers (8 female, age range: 20–35) participated in Experiment 2. We used the detection task in Experiment 2, in which catch trials (no target trials) were included. Each catch trial ended 1 s after eye gaze moved to left/right. A total of 1,134 trials included 126 catch trials (about 11.1%). Participants were instructed to press the space key only when the target Gabor was presented. If participants did not press a key in 1 s after a target appeared, such trials were considered miss trials.

### Results

**Figure 3:**
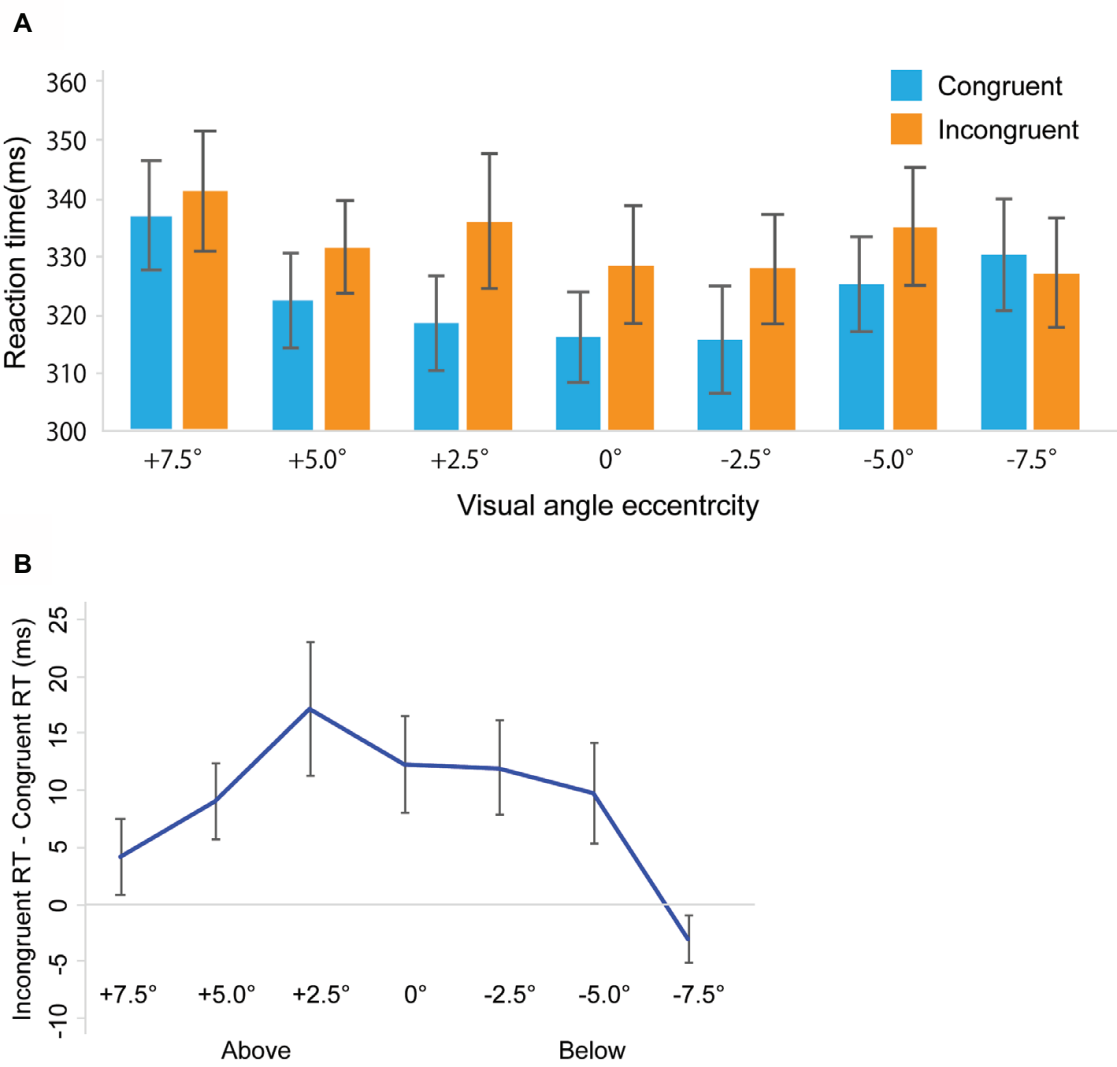
Results in Experiment 2. **(A)** Mean of (individual) median reaction time (ms). **(B)** The results of the magnitude of the gaze cuing effect (incongruent RT-congruent RT). The error bars represent standard error of mean.

Miss trials were removed from further analyses (Total: 4.82%, congruent trials: 5.02%, incongruent trials: 4.61%). False alarm rates in the catch trials were 8.6%. Figure 3A shows RTs in the gaze cuing task, and Figure 3B shows gaze cuing effects (RTs in the incongruent-congruent conditions) on RTs. We performed a repeated measures ANOVA based on a 2 *Congruency* (congruent, incongruent) × 7 *Location* (+7.5, +5.0, 2.5, 0, −2.5, −5.0, −7.5°) design. The main effects of both *Congruency* [*F*_(1, 20)_ = 24.032, *p* < 0.001, $\eta_{p}^{2}$ = 0.545] and *Location* [*F*_(1, 20)_ = 9.115, *p* < 0.001, $\eta_{p}^{2}$ = 0.313] emerged. Also, a significant interaction between *Congruency* and *Location* [*F*_(1, 20)_ = 2.561, *p* < 0.05, $\eta_{p}^{2}$ = 0.113] was evident. To further assess this interaction between *Congruency* and *Location*, a simple main effect analysis was performed with a Bonferroni correction. This analysis yielded significant differences between congruent and incongruent conditions in the +5.0° (*t*_20_ = 2.635, *p* < 0.05, *d* = 0.24), +2.5° (*t*_20_ = 2.821, *p* < 0.05, *d* = 0.37), 0° (*t*_20_ = 2.841, *p* < 0.05, *d* = 0.29), −2.5° (*t*_20_ = 2.831, *p* < 0.05, *d* = 0.28), and −5° conditions (*t*_20_ = 2.165, *p* < 0.05, *d* = 0.23). Furthermore, we found a simple main effect of *Location* in the congruent condition [*F*_(1, 6)_ = 8.535, *p* < 0.001, $\eta_{p}^{2}$ = 0.299] and incongruent conditions [*F*_(1, 6)_ = 3.104, *p* < 0.01, $\eta_{p}^{2}$ = 0.13].

### Discussion

In Experiment 2, using the detection task, we observed results that were qualitatively similar to results in Experiment 1. Thus, the results in Experiment 1 should not be attributed to the occurrence of stimulus-response compatibility.

In Experiments 1 and 2, we did not find gaze cuing effects in the ±7.5° conditions. Considering the effective field of view regarding gaze cuing effects, two factors should be examined: one involves spatial resolutions in peripheral vision, and the other concerns gaps between gaze directions of the facial image and target locations. Spatial resolutions decrease as eccentricities increase (Rosenholtz, 2016), and the cortical representations are larger in foveal than in peripheral vision (Rovamo and Virsu, 1979). Therefore, if the size of the facial image is adjusted to reflect cortical representations, then the effective field of view regarding gaze cuing effects might be enlarged for a participant. Concerning gaps between gaze directions and target locations, the eyes of facial images in Experiments 1 and 2 were not oriented toward possible target locations precisely when the facial image was presented in peripherally. For example, although a target appeared at an obliquely downward (upward) location from a face image in the congruent with +7.5° (−7.5°) condition, the eye gaze of the image was directed to horizontal locations. Thus, if the facial image accurately looked at the possible target location, gaze cuing effects might be stronger. In Experiment 3, we manipulated the image size and gaze directions of the facial images based on eccentricities. The goal was to assess whether the effective field of views regarding gaze cuing effects would be enlarged.

## Experiment 3

We conducted two experiments in Experiment 3. These involved localization and detection tasks in Experiments 3a and 3b, respectively.

### Method

The method in Experiments 3a (localization task) and 3b (detection task) was identical to Experiments 1 and 2, respectively, with the exception of the following details.

### Participants and Visual Stimuli

**Figure 4:**
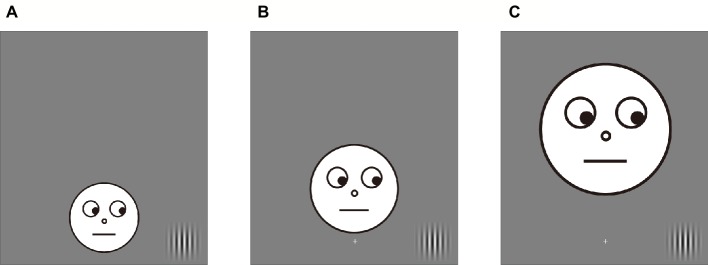
Examples of facial stimuli in Experiment 3. Eyes of the facial image accurately looked at the possible target locations. **(A)** An example of the +2.5° condition. **(B)** An example of the +5.0° condition. **(C)** An example of the +7.5° condition.

Nineteen paid volunteers participated in Experiment 3a (6 female, age range: 20–33); the same number of paid volunteers participated in Experiment 3b (10 female, age range: 20–29). We calculated the M (a cortical magnification factor) value based on the study of Rovamo and Virsu (1979) to adjust the facial images in peripheral vision. As a result, size of the facial images was 4.08° × 4.08°, 5.87° × 5.87°, and 7.68° × 7.68° in the ±2.5, ±5.0, and ±7.5° conditions, respectively (the image size of the 0° condition was 2.3° × 2.3°). In addition, eye gaze of the facial images was oriented toward the possible target locations in all location conditions (Figure 4).

### Results

#### Experiment 3a (Localization Task)

**Figure 5:**
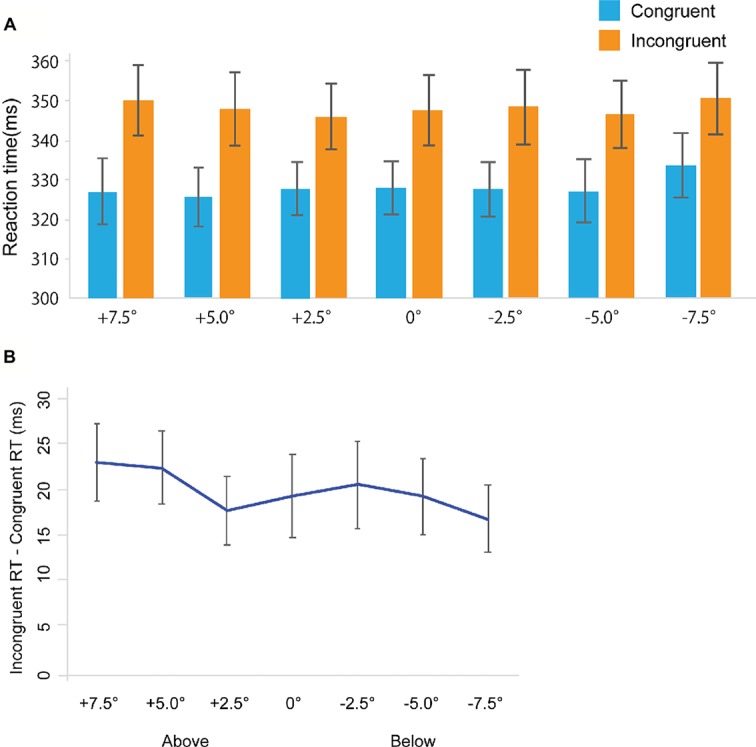
Results in Experiment 3a. **(A)** Mean of (individual) median reaction time (ms). **(B)** The results of the magnitude of the gaze cuing effect (incongruent RT-congruent RT). The error bars represent standard error of mean.

Error trials were removed from further analysis (Total: 2.56%, congruent trials: 1.85%, incongruent trials: 3.27%). Figure 5A shows RTs in the gaze cuing task, and Figure 5B shows gaze cuing effects (RTs in the incongruent-congruent conditions) on RTs. We performed 2 *Congruency* (congruent, incongruent) × 7 *Location* (+7.5, +5.0, 2.5, 0, −2.5, −5.0, −7.5°) repeated measures ANOVA. We found the main effect of *Congruency* [*F*_(1, 18)_ = 31.036, *p* < 0.001, $\eta_{p}^{2}$ = 0.633]. However, we did not observe a main effect of *Location* [*F*_(1, 18)_ = 1.862, *p* = 0.094, $\eta_{p}^{2}$ = 0.093]; also, no significant interaction between *Congruency* and *Location* was observed [*F*_(1, 18)_ = 0.790, *p* = 0.579, $\eta_{p}^{2}$ = 0.042].

#### Experiment 3b (Detection Task)

**Figure 6:**
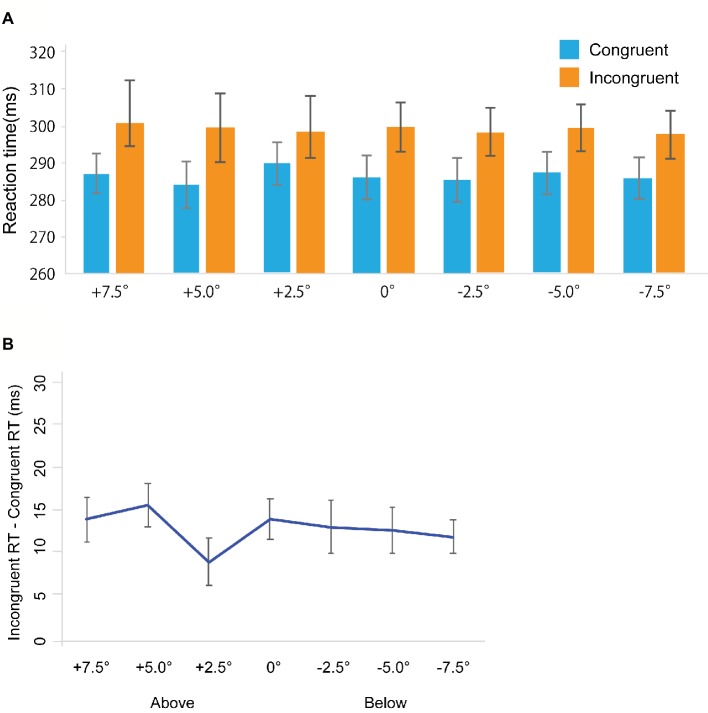
Results in Experiment 3b. **(A)** Mean of (individual) median reaction time (ms). **(B)** The results of the magnitude of the gaze cuing effect (incongruent RT-congruent RT). The error bars represent standard error of mean.

Miss trials were removed from further analysis (Total: 1.38%, congruent trials: 1.54%, incongruent trials: 1.23%). False alarm rates in the catch trials were 6.3%. Figure 6A shows RTs in the gaze cuing task, and Figure 6B shows gaze cuing effects (RTs in the incongruent-congruent conditions) on RTs. We performed a repeated measures ANOVA with 2 *Congruency* (congruent, incongruent) × 7 *Location* (+7.5, +5.0, +2.5, 0, −2.5, −5.0, −7.5°). We found a significant main effect of *Congruency* [*F*_(1, 18)_ = 53.369, *p* < 0.001, $\eta_{p}^{2}$ = 0.748]. However, a main effect of *Location* was not significant [*F*_(1, 18)_ = 0.712, *p* = 0.640, $\eta_{p}^{2}$ = 0.038]; also, no significant interaction between *Congruency* and *Location* was observed [*F*_(1, 18)_ = 0.936, *p* = 0.472, $\eta_{p}^{2}$ = 0.049].

### Discussion

In Experiment 3, we manipulated size of the facial images based on the cortical representations and accurate gaze directions of facial images. In the both experiments, we observed gaze cuing effects in all the location conditions. However, we did not find interaction between congruency and location that we observed in Experiments 1 and 2. Hence, our manipulations in Experiment 3 enlarged the effective field of view regarding gaze cuing effects.

## General Discussion

The present study provides the first evidence of gaze cuing effects in peripheral vision. We also found that the gaze cuing effects persist up to ±5° of vertical eccentricity when we did not manipulate size of facial stimuli and gaze directions (eye gaze directed horizontally), regardless of eccentricities (Experiments 1 and 2). Previous studies have indicated that accurate gaze discrimination is up to 4–6° of the visual angles (Loomis et al., 2008; Palanica and Itier, 2014, 2017). Note that, in vertical eccentricity, Palanica and Itier (2017) reported that accurate gaze discrimination can occur up to ±6° of the visual angle. Our results are in a range of these findings in Palanica and Itier (2017). Thus, gaze perception, including gaze cuing effects, should occur up to approximately ±6° of vertical eccentricities (but see Florey et al., 2015).

Based on the results in Experiments 1 and 2, peripheral vision is more likely to influence the congruent condition rather than the incongruent condition. We observed a simple main effect of *Location* in the congruent condition in both Experiments 1 and 2. Although we observed a simple main effect of *Location* in the incongruent conditions in Experiment 2 (but not in Experiment 1), the effect size of the incongruent condition was much smaller than that in the congruent condition (congruent condition: $\eta_{p}^{2}$ = 0.299, incongruent condition: $\eta_{p}^{2}$ = 0.13). Hence, periphery appears to impact attentional benefits (rather than attentional costs) by perception of gaze direction. Although gaze cuing effects exist in the peripheral vision, attentional benefits gained by perception of gaze direction are stronger in the foveal vision than in peripheral vision.

If image size changed corresponding to the cortical representations and its eyes, i.e., in a relevant facial image, are appropriately oriented toward the possible target location (Experiment 3), then the effective field of view regarding gaze cuing effects is enlarged. In addition, because we did not find an interaction between congruency and face locations in the experiment, it is plausible that certain eccentricities, namely those with visual angles up to ±7.5°, do not influence gaze cuing effects given such manipulations. It should be noted that Florey et al. (2015) have proposed that reduced spatial resolution in peripheral vision might not lead to a decrease of accuracy in a gaze discrimination task when participants are required to identify the gaze directions of face stimuli. This may be inconsistent with the present findings; that is, manipulation of face size in peripheral conditions could enlarge the effective field of view regarding gaze cuing effects. Although speculative, this inconsistency may be caused by the differences in cognitive processes. When gaze cuing effects are induced, participants have to perceive gaze direction of the facial image and then shift their attention to the location implicated the eye gaze. On the other hand, the gaze discrimination task required only the perception of gaze directions. Thus, it is possible that the lack of a gaze cuing effect in the ±7.5° condition of Experiments 1 and 2 is caused by a dysfunction of attentional shifts induced by lower accuracy of face/gaze representations. Further studies are needed to clarify this issue.

In conclusion, we have shown gaze cuing effects exist outside foveal vision. Gaze perception plays an important role in social interactions, and when we interact within a group, gaze perception beyond foveal vision is critical. Gaze cuing effects occur in periphery, and this social ability helps us to engage in smooth social interactions with others.

## Author Contributions

TY conceived the experiments. TY and YT designed the experiments. TY performed the experiments. TY and YT analyzed the data, contributed reagents, materials and analysis tools, and wrote the main manuscript text. TY prepared figures.

### Conflict of Interest Statement

The authors declare that the research was conducted in the absence of any commercial or financial relationships that could be construed as a potential conflict of interest.
